# The regulation of immune cells by Lactobacilli: a potential therapeutic target for anti-atherosclerosis therapy

**DOI:** 10.18632/oncotarget.18346

**Published:** 2017-06-02

**Authors:** Ya-Hui Ding, Lin-Yan Qian, Jie Pang, Jing-Yang Lin, Qiang Xu, Li-Hong Wang, Dong-Sheng Huang, Hai Zou

**Affiliations:** ^1^ Department of Cardiology, Zhejiang Provincial People's Hospital, Hangzhou 310014, Chinaa; ^2^ People's Hospital of Hangzhou Medical College, Hangzhou 310014, China; ^3^ Department of Hepatobiliary Surgery, Zhejiang Provincial People's Hospital, Hangzhou 310000, China

**Keywords:** atherosclerosis, Lactobacillus, lymphocyte, macrophage, dendritic cell

## Abstract

Atherosclerosis is an inflammatory disease regulated by several immune cells including lymphocytes, macrophages and dendritic cells. Gut probiotic bacteria like *Lactobacilli* have been shown immunomodificatory effects in the progression of atherogenesis. Some *Lactobacillus* stains can upregulate the activity of regulatory T-lymphocytes, suppress T-lymphocyte helper (Th) cells Th1, Th17, alter the Th1/Th2 ratio, influence the subsets ratio of M1/M2 macrophages, inhibit foam cell formation by suppressing macrophage phagocytosis of oxidized low-density lipoprotein, block the activation of the immune system with dendritic cells, which are expected to suppress the atherosclerosis-related inflammation. However, various strains can have various effects on inflammation. Some other *Lactobacillus* strains were found have potential pro-atherogenic effect through promote Th1 cell activity, increase pro-inflammatory cytokines levels as well as decrease anti-inflammatory cytokines levels. Thus, identifying the appropriate strains is essential to the therapeutic potential of *Lactobacilli* as an anti-atherosclerotic therapy.

## INTRODUCTION

Atherosclerosis (AS) is one of the most common chronic non-infectious diseases worldwide. AS is associated with several serious cerebrovascular diseases such as acute coronary syndrome, and stroke. AS was originally thought to result from accumulation of toxic lipids due to dysfunctional lipid metabolism. In recent years, however, mounting evidence suggests that inflammation, which involves innate and adaptive immune responses, may play a critical role in the development and progression of AS. For example, large concentrations of immune cells, such as macrophages, dendritic cells (DCs), and lymphocytes, have been identified in AS plaques. These cells secrete chemokines and pro-inflammatory, anti-inflammatory factors and adhesion factors, which contribute to AS pathogenesis [1].

This revelation suggests that anti-inflammatory therapeutics may inhibit or reverse the progression of AS. In a recent study, it was determined that treatment with anti-inflammatory pharmaceuticals statins reduced incidents of stroke in a rat model of AS [2]. Inhibitors of renin-angiotensin system (RAS), calcium channel blockers, aspirin and some other medicines also exhibited anti-atherogenetic effects by reducing inflammation.

A recent anti-inflammatory agent of interest is probiotic bacteria *Lactobacilli*. This probiotic is essential to several bodily processes including fermentation and decomposition of indigestible substances, stimulation of cell growth, regulation of the immune system, and destruction of pathogenic bacteria. Several recent studies have revealed a potential therapeutic role for probiotic bacteria against AS. Recent studies found that some *Lactobacillus* strains significantly reduce the arteriosclerotic index [3–5]. These effects are largely due to the immunomodulatory functions of *Lactobacilli*. Remarkably, the difference of immune modification effects in various strains of *Lactobacilli* leads to distinguished features in pathogenesis of atherosclerosis. Some strains of *Lactobacilli* promote the inflammatory response of immune cells which may augment atherogenesis [6–9]. In this review, we will provide an overview of the effects concerning AS, especially the potential anti-atherogenetic effects, of *Lactobacilli* mediated through immune cells.

### Overview of immune cells in the development and progression of AS

**Figure 1:**
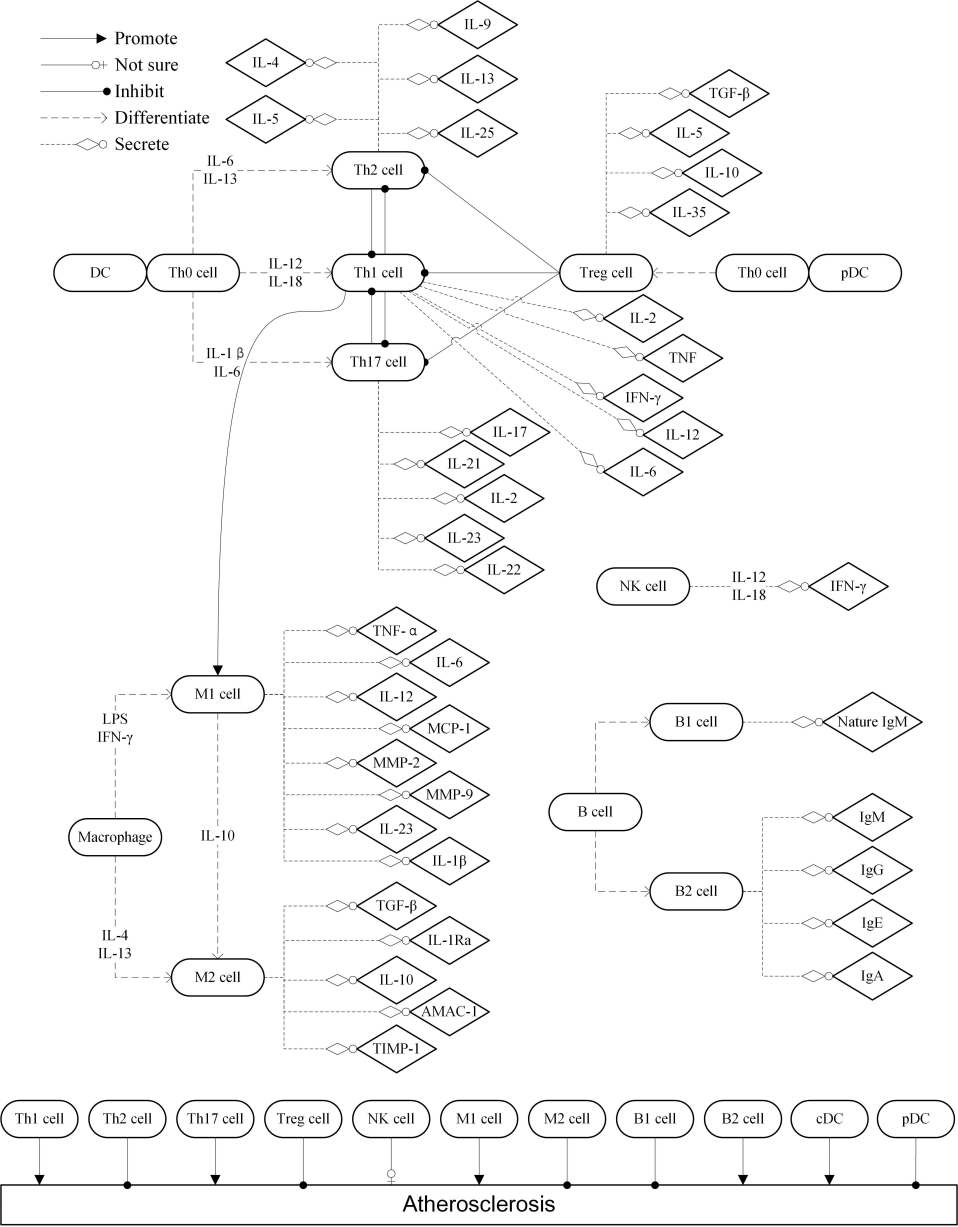
The role of immune cells in the progression of atherogenesis DC: dendritic cell; IL: interleukin; TNF: tumor necrosis factor; IFN: interferon; TGF: transforming growth factor; Treg: regulatory T; Th: T-lymphocyte helper; pDC: plasmacytoid DC; cDC: classic DC; MCP: monocyte chemotactic protein; AMAC: alternative macrophage activation-associated CC chemokine; TIMP: tissue inhibitor of metalloproteinase; NK: nature killer; MMP: metal matrix proteinase.

AS involves the complicated interaction between several immune cells and cytokines, its triggering factors include lipoprotein, reactive oxygen species, hypertension, shear force, smoking, etc. Endothelial dysfunction induces the initiation of atherogenesis which characterized by chemotaxis and adhesion of monocytes and T-lymphocytes to the endothelial surface via chemotactic factor like MCP-1 and adhesion molecules like VCAM-1. Then, various immune cells involve in the progression of atherogenesis (Figure 1).

### Monocytes and macrophages

Macrophages are important immune cells in both innate and adaptive immune responses. They are also an important source of inflammatory factors. Macrophages play a critical role in the development of AS. During AS progression, the macrophages aggregate to form “foam cells” which increase the rupture risk of AS plaques [10, 11].

The monocytes migrate into the arterial intima and, in response to chemokines and related receptors, convert into macrophages driven by cytokines like macrophage colony-stimulating factor (M-CSF), and express Toll-like receptor (TLR), pattern recognize receptor (PRR) and scavenger receptor [12, 13]. Macrophages take up low-density lipoprotein (ox-LDL) through scavenger receptor B, and in response release pro-inflammatory cytokines which promotes a focal arterial endothelial immune response and accelerates the formation and development of AS [14]. Myeloid monocytes differentiate into pro-inflammatory (M1) and anti-inflammatory (M2) macrophages after migrating into tissues. M1 macrophages kill microbes and produce pro-inflammatory cytokines, such as tumor necrosis factor α (TNF-α), interleukin 6 (IL-6), IL-12 and MCP-1, as well as secrete extracellular matrix proteins, MMP-2 and MMP-9, all of which exacerbate AS. M2 macrophages produce anti-inflammatory cytokines, such as IL-10, TGF-β, IL-1Ra and AMAC-1 (CCL-18) while also removing cell fragments, promoting angiogenesis and improving tissue remodeling and repair [15]. Differentiated M1 and M2 macrophages can be converted to one another [16]. M2 macrophages convert to M1 macrophages during plaque progression while M1 macrophages convert to M2 macrophages during plaque regression [17]. Thus, the polarization of macrophages may serve as biomarkers of the pathologic progression of AS in principle.

### T-lymphocytes

T-lymphocytes play a key role in the development and progression of AS. Following the formation of AS plaques, T-lymphocytes cluster along the periphery, fibrous cap and in the center of the plaques. As the disease progresses, the number of bordering T-lymphocytes gradually increases. The T-lymphocytes are activated by various endogenous and exogenous stimulators, such as ox-LDL. The activated T-lymphocytes then secrete cytokines like granulocyte-macrophage colony stimulating factor (GM-CSF), interferon γ (IFN-γ), TNF-β, IL-2, IL-4 and IL-6, which promote the development of AS [1]. T-lymphocytes can be classified into subsets according to their immunophenotype. These subsets include helper T (Th) cells and regulatory T (Treg) cells. Th1 cells promote inflammatory responses by secreting pro-inflammatory cytokines, like IFN-γ, TNF-α and TNF-β [18]. In AS plaques Th1 cells produce IFN-γ and activate macrophages [19]. Th2 cells can inhibit macrophages phagocytize ox-LDL mediated by scavenger receptor. Deletion of IL-5 and IL-13, two Th2 cytokines, accelerates AS [20, 21], while deficiency of IL-4, another Th2 cytokines may attenuate the development of AS [22]. So the anti-atherogenetic effects of Th2 cells are still unclear and controversial [23]. Th17 cells mainly secrete inflammatory cytokine like IL-17 and IL-2. A proatherogenic role of IL-17 is found in some studies, but the results are controversial [24]. IL-17 blockade led to reduce AS in Apoe−/− mice in some studies [25, 26], while in other study, IL-17 did not affect plaque burden even it contributes to vascular and systemic inflammation [27]. Furthermore, the increased number of Th17 cells or Th17/Treg ratio were found in some clinical research [28, 29]. It suggests that Th17 cells have a detrimental impact on atherosclerotic plaque stability. Treg cells inhibit the immune response of DC, Th1, Th2 and Th17, and increase the expression of TGF-β, IL-10, and IL-5, which may inhibit the proliferation of bystander T cells in an IL-10-dependent fashion [30, 31].

### Nature killer cells

Nature killer (NK) cells, innate lymphocytes capable of lysing target cells, have an immunoregulatory effects in the pathogensis and development of AS. MCP-1 recruits NK cells in the AS lesions, and CX3CL1 induce NK cell migration and activation which cause increased cytotoxicity and pro-atherogenic cytokine IFN-γ [32, 33]. Increased circulating NK cell levels were found in some studies [34, 35], however, reduced NK cell levels were observed in other studies [36–38]. A study in LDLR−/− beige mice suggested that atheroprotective effect of NK cells was independent of its cytotoxicity but cytokine production might be the major factor [39]. Therefore, it is still unclear whether NK cells are pro-atherogenesis or anti-atherogenesis, related to their cytolytic activity or cytokine secretion.

### B-lymphocyte

B-lymphocytes, derived from bone marrow, are vital to adaptive immunity in that they by produce immunoglobulin that participate in humoral immunity. According to recent studies, the immune-protective effect of B-lymphocytes may also contribute to AS development [40, 41]. However, the lack of B-lymphocytes promotes the formation and development of coronary atherosclerosis heart diseases in human [42]. Further research revealed the different anti-atherogenetic effects of various B-lymphocytes subsets. B1 cells prevent lesion formation, whereas B2 cells promote it [43]. B1 cells secrete poorly specific nature IgM antibodies and attenuate atherosclerotic burden, which may link with preventing oxLDL internalization by macrophages and apoptotic cell accumulation by enhanced efferocytosis [43, 44]. B2 cells secrete all human immunoglobulin classes, namely IgM, IgG, IgE and IgA, and appear to augment atherogenesis through antibody dependent or independent mechanisms [45, 46].

### Dendritic cells

Dendritic cells (DCs) are the most powerful professional antigen-presenting cells (APC). DCs are subdivided into plasmacytoid DCs (pDCs) and classic DCs (cDCs) depending on their phenotype and functions. DCs take in and process antigens effectively, then present antigens to memory T-lymphocytes to activate an immune response. DCs can secrete several cytokines, mainly IL-12, IL-10, IL-23, IL-6, IL-1β, TNF-α, TGF-β, as well as express various maturation markers, such as CD40, CD54, CD80, CD83, CD86 and MHC complex [47, 48]. Activated DCs in the vascular endothelium highly expressed adhesion markers like ICAM-1, and VCAM-1 which activate T-lymphocytes [2]. Lord et al. found that DC-mediated immune responses were found to be involved in the early stage of atherogenesis, evidenced by the fact that DCs increased significantly in the athero-prone areas of normal arterial wall [49–52]. Moreover, high expression of DC's costimulate molecule CD86 is associated with stable coronary artery disease as well as acute coronary syndrome [53]. Some DC subsets, such as Intestinal CD103+ DCs, are also the key for tolerogenic immune responses, they can promote the differentiation of Treg cells [54, 55]. Manikandan et al. found that MyD88 signaling in CD11C+ DCs play a key role during the T-lymphocytes activation in atherogenesis because it promote the development of atheroprotective Treg cells [56]. Furthermore, Isabelle et al. found that pDCs demonstrated a protective role in AS [57]. It may be caused by inhibiting T cell proliferation and activity in peripheral lymphoid tissue. Thus suppressing DC maturity and antigen-present function is regarded as one of the anti-atherogenetic mechanisms of statins [2].

### Inflammatory response of *Lactobacilli* in lymphocytes

### T-lymphocyte

Several studies now show that *Lactobacilli* reduces inflammatory response via T-lymphocytes. Particularly, Treg cells play a vital role in the inhibitory effect of *Lactobacilli* on inflammation. There are positive correlations between the number of Treg cells and *Lactobacilli* [30, 58–62]. Treg cells increase IL-10 level and inhibit the proliferation of bystander T cells in an IL-10-dependent fashion [30]. *Lactobacilli* are also effective in inducing CD4+CD25+Foxp3+ Treg cell mediated tolerance [63].

In Kim et al. study, treatment with *Lactobacillus rhamnosus* Lcr35 was found to increase the number of CD4+CD25+Foxp3+ Treg cells in the spleens and mesenteric lymph nodes of mice [59, 60, 64]. Lcr35 also suppressed Th1 (IFN-γ), Th2 (IL-4, IL-5, and IL-13) and Th17 (IL-17) cell cytokines in the serum, and thymic stromal lymphopoietin (TSLP) responses. The protective effects of Lcr35 was blocked by anti-CD25 mouse antibody, which indicated that CD4+CD25+Foxp3+ Treg cells are indispensable in mediating the activity of Lcr35. Similar effects, (ie upregulation of CD4+CD25+Foxp3+ Treg cells) was observed with other *Lactobacillus* strains, such as *Lactobacillus rhamnosus* GG [65], *Lactobacillus casei* ATCC 334 [66], *Lactobacillus reuteri* ATCC 23272 [58], and *Lactobacillus paracasei* L9 [63]. Furthermore, Reynolds et al. found that *Lactobacillus taiwanesis* elevated Treg cells in the gut-associated lymphoid tissue without raising Th17 cell responsiveness [62].

However, it is important to note that different strains may have different immunomodulatory effects on Th1 and Th2 cells. A study in healthy wild-type male BALB/c mice showed that, in the small intestinal lamina propria, *Lactobacillus plantarum* WCFS1 significantly decreased the Th1/Th2 cell ratio. However, *Lactobacillus salivarius* UCC118 and *Lactobacillus lactis* MG1363 had no effect [67]. *Lactobacillus rhamnosus* LA68, on the other hand, activated the Th1 immune response in healthy C57BL/6 mice [6]. Studies on hypersensitive ovalbumin (OVA)-sensitized mice/rats revealed that *Lactobacillus* strains induced Th1 cytokines and inhibit Th2 cell cytokines, which improved immune balance and relieved the hypersensitivity [7–9]. Similar changes to the Th1/Th2 cell ratio were observed in influenza A/NWS/33 (H1N1) virus (IFV) infected BALB/c mice that were administrated by *Lactobacillus fermentum* CJL-112 [68]. Moreover, in the same mouse model, *Lactobacillus casei* Shirota attenuated the Th2 cell phenotype. In contrast, *Lactobacillus plantarum* WCFS1 augmented the Th2 cell phenotype [69]. Taken together, these studies demonstrate that the variant between *Lactobacillus* strains plays a critical role in immune response variation (Table 1).

**Table 1:** Inflammatory response of Lactobacilli in T-lymphocytes

Strains	Th1	Th2	Th17	Treg	Inflammatory cytokines	Reference
Lactobacillus rhamnosus Lcr35	↓	↓	↓	↑	IL-4↓, IL-17↓, IFN-γ↓, IL-5↓, IL-13↓	Jang et al. [59, 60, 64]
Lactobacillus rhamnosus GG				↑	IL-10↑, IL-6↓	Khailova [65]
Lactobacillus casei ATCC 334				↑	IL-10↑	Tiittanen [66]
Lactobacillus reuteri ATCC 23272				↑	IL-10↑, MCP-1/CCL2↓, TNF↓, IL-5↓	Karimi [58]
Lactobacillus paracasei L9				↑	IL-10↑, TGF-β↑, IFN-γ↑, IL-4↓	Yang [63]
Lactobacillus taiwanesis				↑		Reynolds [62]
Lactobacillus plantarum WCFS1	↓	↑			IL-4↑	Meijerink et al. [67, 69]
Lactobacillus salivarius UCC118				↑		Smelt [67]
Lactobacillus rhamnosus LA68	↑				IFN-γ↑, IL-10↓	Dimitrijevic [6]
Lactobacillus plantarum CJLP133	↑				IL-10↑, IL-12↑, IFN-γ↑, IL-4↓	Won [8]
Lactobacillus brevis HY7401	↑				IFN-γ↑, IL-12↑, IL-4↑, IL-5↑, IL-6↓, IL-10↓	Lee [7]
Lactobacillus rhamnosus GG ATCC 53103 & Bifidobacterium longum BB536	↑				IFN-γ↓, IL-4↓, IL-10↓	Huang [9]
Lactobacillus fermentum CJL-112	↑	↓			IFN-γ↑, IL-2↑, IL-4↓, IL-5↓, IL-10↓	Yeo [68]
Lactobacillus salivarius HMI001 & Lactobacillus casei Shirota		↓			IL-4↓, IL-5↓	Meijerink [69]
Lactobacillus plantarum nF1	↑	↓	↑		TNF-α↑, IL-12 p70↑, IL-4↓, IL-5↓, IL-6↑, IL-17A↑	Lee [89]
Lactobacillus rhamnosus MTCC 5897	↑	↓			IL-4↓, IFN-γ↑	Saliganti [106]
Lactobacillus plantarum K8	↑				IL-12↑, IFN-γ↑, IL-4↓	Kim [107]

MCP: monocyte chemotactic protein; IL: interleukin; G-CSF: granulocyte-colony stimulating factor; TNF: tumor necrosis factor; IFN: interferon; TGF: transforming growth factor; CCL: C-C chemokine ligand.

Generally, *Lactobacilli* can be roughly classified as pro-inflammatory strains and anti-inflammatory strains according to their influence on T-lymphocyte subsets. But the effects of some T-lymphocyte subsets like Th2 and Th17 are still controversial as well as the complex interaction between T-lymphocyte subsets, it is difficult to explicate the anti-atherogenetic effects of *Lactobacilli* via T-lymphocytes path exactly. Nevertheless, current research revealed that Treg cells are a key component in inhibiting AS-related inflammation. Certainly, the exact roles of T-lymphocytes in the formation and development of AS are still need to be elucidated.

### B-lymphocyte

The Lee et al. study showed *Lactobacillus plantarum* could stimulate murine splenocyte proliferation, and this effect in dead nano-sized *Lactobacillus plantarum* was more apparent than in pure live bacteria [7]. *Lactobacillus helveticus* SBT2171 was found to inhibit the proliferation of T-lymphocytes and B-lymphocytes in LPS-stimulated mice [70]. While, *Lactobacillus plantarum* CJNR26 and *Lactobacillus gasseri* CJMF3 increased the B-lymphocyte population in the spleen of mice [71]. A dose-response, double-blind, placebo-controlled, randomized pilot trial showed that low dose *Lactobacillus plantarum* CECT 7315 and CECT 7316 increased activated B-lymphocytes (CD19+) as well as T-helper lymphocytes (CD4+CD25+) [72]. But unfortunately, until recently, there was no direct evidence demonstrating the anti-atherogenetic effect of *Lactobacilli* via B-lymphocytes. Therefore, it is unclear what role *Lactobacilli* are playing in the atherogenesis and development of AS via B-lymphocyte pathway.

### Nature killer cells

Some studies showed that *Lactobacilli* can augment NK cell activity, like *Lactobacillus plantarum* 06CC2 [73], *Lactobacillus delbrueckii* OLL1073R-1 [74], *Lactobacillus casei* Shirota [75], Lactobacillus casei HY7213 [76]. Lee et al. found that *Lactobacillus plantarum* HY7712 protected against the impairment of NK cell activity caused by γ-irradiation or aging through activating the TLR2/NF-κB signaling pathway (24105270). In fact, *Lactobacilli* can activate NK cells as well as induce augmentation of immune responses of Th1 cells, cytotoxic T cells, macrophages [73, 76], increase the production of IFN-γ, IL-12 [74, 77, 78] at the same time. However, Dong et al. found *Lactobacillus casei* Shirota improved NK cell activity as well as increase IL-10/IL-12 ratio in older population [79]. Another study involved six probiotic strains (*Lactobacillus casei* Shirota, *Lactobacillus rhamnosus* GG, *Lactobacillus plantarum* NCIMB 8826 and *Lactobacillus reuteri* NCIMB 11951, *Bifidobacterium longum* SP 07/3 and B. bifidum MF 20/5) showed increasing NK cell activity, but some cytokines levels, like IL-10, IFN-γ, IL-12p70, IL-6 and MCP-1, were strain-specific [80].

The truth is, there are still much controversy to the effects of NK cells in atherogenesis, so it is still unknown about NK cell activation by *Lactobacilli* for atherogenesis and development of AS unless there is some direct evidence.

### Inflammatory response of *Lactobacilli* in macrophage

A study found administration of *Lactobacillus gasseri* SBT2055 decreased the number of macrophages and the M1/M2 ratio in mice [81]. The similar effects could be observed in *Lactobacillus paracasei* [82], *Lactobacillus plantarum* CLP-0611 [83], and *Lactobacillus brevis* G-101 [84]. Furthermore, some *Lactobacillus* strains, such as *Lactobacillus plantarum* OLL2712 [85], *Lactobacillus rhamnosus* ATCC 7469 [86], *Lactobacillus rhamnosus* GG MTCC 1408 [87] and *Lactobacillus helveticus* NS8 [88] were revealed to increase the production level of IL-10 which is regarded as an important anti-inflammatory cytokine that inhibits atherogenesis.

On the other hand, some *Lactobacillus* strains were found to increase the pro-inflammatory production levels of macrophages, like TNF-α and IL-6 [86, 89, 90]. However, decreased TNF-α and IL-6 production levels of macrophages were observed after treatment with other *Lactobacillus* strains [81, 87, 91]. Taken together, these results indicate that the pro-inflammatory and anti-inflammatory effects of *Lactobacilli* have notable differences in various strains and experimental models.

In fact, Lactobacilli have influenced cholesterol metabolism in macrophages. *Lactobacillus paracasei* regulate alveolar macrophages cholesterol metabolism and the response to LPS in Ossabaw pigs. It decreased the concentrations of cholesteryl-esters and suppressed expression of pro-inflammatory mediators in alveolar macrophages [92]. In some clinical trials, such effects are also found, but the results are somewhat ambiguous. A controlled, randomized, double-blind trial discovered administration of *Lactobacillus plantarum* mixture of three strains (CECT 7527, CECT 7528 and CECT 7529) reduced the plasma cholesterol levels with ox-LDL in hypercholesterolaemic patients [93]. *Lactobacillus delbrueckii* bulgaricus 2038 was also found to reduce LDL oxidation in F344 rats [94]. But another randomized, double-blind intervention in marathon runners revealed that *Lactobacillus rhamnosus* GG had no effect on regulation of ox-LDL, s-TRAP or serum antioxidants levels during the study [95]. However, a clinical trial in patients over 65 years old found that the ox-LDL level is inversely proportional to the number of *Lactobacilli* [96]. In addition, Yoon et al. found that *Lactobacillus rhamnosus* BFE5264 and *Lactobacillus plantarum* NR74 may block foam cell formation by cholesterol efflux and immune modulation in THP-1 macrophage cells [97].

Therefore, like the lymphocytes, inflammatory response of macrophages is different in various *Lactobacillus* strains, and it is vital that the appropriate strains are used to study anti-atherogenetic effects (Table 2).

**Table 2:** Inflammatory response of Lactobacilli in macrophages

Strains	Macrophage number	M1/M2 ratio	Cholesterol	Inflammatory cytokines or biochemical markers	Reference
Lactobacillus gasseri SBT2055	↓	↓		CCL2↓, CCR2↓, LEP↓	Ukibe [81]
Lactobacillus paracasei LPC4	↓	↓		TLR-4↓, NOX-4↓, TNF-α↓, MCP-1↓, IL-4↓,, PPAR-γ↓, PPAR-δ↓,	Sohn [82]
Lactobacillus plantarum CLP-0611		↓		IL-1β↓, IL-6↓, NF-κB↓, AP1↓, IL-10↑, CD206↑	Jang [83]
Lactobacillus brevis G-101		↓		IL-10↑, IL-1β↓, IL-6↓, TNF-α↓, NF-κB↓	Jang [84]
Lactobacillus plantarum OLL2712				IL-10↑, IL-6↓, TNF-α↓, MCP-1↓	Toshimitsu [85]
Lactobacillus rhamnosus ATCC 7469				TNF-α↑, IL-6↑, IL-10↑, IL-12↓	Jorjao [86]
Lactobacillus rhamnosus GG MTCC 1408				IL-10↑, TNF-α↓	Divyashri [87]
Lactobacillus helveticus NS8				IL-10↑	Rong [88]
Lactobacillus plantarum nF1				TNF-α↑, IL-12 p70↑, IL-6↑, IL-17↑, IL-4↓	Lee [89]
Lactobacillus acidophilus JTB05				IFN-γ↑	Quinteiro-Filho [90]
Lactobacillus salivarius JTB07				IFN-γ↑, IL-1β↑, IL-6↑, IL-8↑, IL-12↑	Quinteiro-Filho [90]
Lactobacillus reuteri JTB07				IL-1β↑, IL-6↑, IL-8↑, IL-12↑	Quinteiro-Filho [90]
Lactobacillus rhamnosus NutRes1				IL-1β↓, IL-6↓, IL-10↓, IL-23↓, TNF-α↓, CXCL-8↓, HMGB1↓	Mortaz [91]
Lactobacillus paracasei			cholesteryl-esters↓	IL-1β↓, IL-8↑, IL-6↑	Trasino [92]
Lactobacillus plantarum mixture (CECT 7527, CECT 7528 and CECT 7529)			TC↓, LDL↓, ox-LDL↓		Fuentes [93][49]
Lactobacillus delbrueckii bulgaricus 2038			ox-LDL↓		Terahara [94]
Intestinal Lactobacillus sp.			ox-LDL↓		Mikelsaar [96]
Lactobacillus rhamnosus BFE5264 and Lactobacillus plantarum NR74			cholesterol efflux↑, foam cells↓	IL-1β↓, TNF-α↓, LXR↑, ABCA1↑, ABCG1↑	Yoon [97]

CCL: C-C chemokine ligand; CCR: C-C chemokine receptor; LEP: leptin; TLR: Toll-like receptor; NOX: NADPH oxidase; TNF: tumor necrosis factor; MCP: monocyte chemotactic protein; IL: interleukin; PPAR: peroxisome proliferator activated receptor; LXR: liver X receptor; ABCA1: ATP-binding cassette transporter A1; ABCG1: ATP-binding cassette transporter G1; CXCL: C-X-C motif chemokine.

### Inflammatory response of *Lactobacilli* in dendritic cells

*Lactobacilli* has a significant influence on DC-related inflammation that are strain dependent (Table 3). For instance, some strains, such as *Lactobacillus casei*, *Lactobacillus acidophilus* NCFM, *Lactobacillus murinus*, and *Lactobacillus salivarius* promote expression of inflammatory cytokines and co-stimulatory molecules more significantly than others, such as *Lactobacillus helveticus* LH-2, and *Lactobacillus acidophilus* La-115 [98, 99]. However, *Lactobacillus plantarum* OLL2712, *Lactobacillus rhamnosus* OLL2838, *Lactobacillus reuteri* 5289, *Lactobacillus paracasei* CBA L74, and *Lactobacillus paracasei* L9 induced anti-inflammatory cytokines in DCs, like IL-10, to exhibit anti-inflammatory effects [63, 85, 100–102]. Furthermore, Zagato et al. found that the suppressive effects of *Lactobacillus paracasei* CBA L74 on inflammation are independent of inactivated bacteria, but respond to metabolic products released during the fermentation process [102].

**Table 3:** Inflammatory response of Lactobacillus in dendritic cells

Strains	Inflammatory cytokines or biochemical markers	Reference
Lactobacillus reuteri 5289	Inhibit Lactobacillus acidophilus NCFM- induced IL-12p70	Amar [101]
Lactobacillus acidophilus NCFM	IL-12p70↑, IL-10↑	Amar [101]
Lactobacillus murinus	IL-10↑, TNF-α↑, IL-6↑, IL-12↑,G-CSF↑,MCP-1↑	Konieczna [98]
Lactobacillus plantarum OLL2712	IL-10↑	Toshimitsu [85]
Lactobacillus rhamnosus OLL2838	IL-10↑, IL-2↑, IL-12↑, TNF-α↑	Ogita [100]
Lactobacillus paracasei CBA L74	IL-10↑	Zagato [102]
Lactobacillus paracasei L9	IL-10↑, TGF-β↑, IFN-γ↑, IL-4↓	Yang [63]
Lactobacillus rhamnosus CRL1505	MHC-II↑	Chiba [103]
Lactobacillus reuteri DSM12246	IL-10↑, inhibit Lactobacillus casei CHCC3137-induced IL-12, IL-6 and TNF-α	Christensen [105]
Lactobacillus casei CHCC3137	IL-10↑, IL-12↑, IL-6↑, TNF-α↑	Christensen [105]
Lactobacillus plantarum Lb1	IL-10↑, IL-12↑, IL-6↑, TNF-α↑	Christensen [105]
Lactobacillus fermentum Lb20	IL-10↑, IL-12↑, IL-6↑, TNF-α↑	Christensen [105]
Lactobacillus plantarum 299v	IL-10↑, IL-12↑, IL-6↑, TNF-α↑	Christensen [105]
Lactobacillus johnsonii La1	IL-10↑, IL-12↑, IL-6↑, TNF-α↑	Christensen [105]
Lactobacillus gasseri SBT2055	TGF-β↑, BAFF↑, IL-10↑, IL-6↑	Sakai [108]
Lactobacillus jensenii TL2937	MHC-II↑, CD80/86↑, IL-10↑	Suda [109]
Lactobacillus rhamnosus CNCM I-4036	IL-1β↑, IL-6↑, IL-8↑, IL-10↑, TNF-α↑	Bermudez-Brito [110]

MCP: monocyte chemotactic protein; IL: interleukin; G-CSF: granulocyte-colony stimulating factor; TNF: tumor necrosis factor; IFN: interferon; TGF: transforming growth factor; MHC: major histocompatibility complex; BAFF: B cell activation factor.

In fact, the influence of inflammatory regulation on DCs by *Lactobacilli* is complicated due to the fact that a single strain can induce pro-inflammatory and anti-inflammatory cytokines simultaneously [103, 104]. Moreover, the immune-modulating properties may rely on the host's genetic background [104]. A study *in vitro* showed that different strains and concentrations of *Lactobacilli* influence factors of DC-related inflammation [105]. After treating DCs with one of three concentrations of *Lactobacilli* (1, 10, and 100 μg/ml), Christensen et al. found that the levels of pro-inflammatory factors, such as IL-12, IL-6 and TNF-α, was highest in ~10 μg/ml while IL-10 was highest in the high bacteria concentration. The study also found that *Lactobacillus reuteri* inhibited *Lactobacillus casei*-induced IL-12, IL-6 and TNF-α production in a dose-dependent manner [105]. *Lactobacillus reuteri* also inhibited the upregulation of CD86 (a co-stimulatory factor that induces T-lymphocyte proliferation and IL-2 production) induced by *Lactobacillus casei*.

## CONCLUSION

Immune cells play a key role in the progression of atherogenesis, which are involved in T-lymphocytes, B-lymphocytes, NK cells, DCs, monocytes/macrophages. *Lactobacilli* are proven regulators of the immune system. Considering the key role of inflammation in atherogenesis and the anti-atherogenetic effect of *Lactobacilli*, immunoregulation for immune cells may be the mechanism by which the probiotic elicits atherogenesis-related effects.

Recent studies found that immunoregulatory effects of *Lactobacilli* are strain-specific (Table 4). Some strains, like *Lactobacillus rhamnosus* Lcr35, decrease Th1 cell number and pro-inflammatory cytokines levels to inhibit the progression of atherogenesis. Other strains, like *Lactobacillus brevis* HY7401, upregulate Th1 cells and promote the secretion of pro-inflammatory cytokines which may accelerate the atherogenesis. The upregulation of Treg cell activity is also an important mechanism of anti-atherogenetic effects of *Lactobacilli*. Serveal strains, such as *Lactobacillus rhamnosus* Lcr35, *Lactobacillus rhamnosus* GG, *Lactobacillus casei* ATCC 334, *Lactobacillus reuteri* ATCC 23272, *Lactobacillus paracasei* L9, etc., are found to increase the Treg cell activity. In addition, inflammatory cytokines secreted by T-lymphocytes are also important part of immune response network. Some strains like *Lactobacillus rhamnosus* LA68 upregulate pro-inflammatory cytokines like IFN-γ as well as downregulate anti-inflammatory cytokines like IL-10. Some other strains like *Lactobacillus casei* ATCC 334 can increase anti-inflammatory cytokines levels. Notably, some strains like *Lactobacillus plantarum* CJLP133 increase pro-inflammatory cytokines and anti-inflammatory cytokines at the same time. So it increase the difficulty to derive the effects of *Lactobacilli* involved in atherogenesis.

**Table 4:** The effects of Lactobacilli in the progression of atherogenesis

Effects	Strains
Anti-atherogenesis	*L. rhamnosus* Lcr35, *L. rhamnosus* GG, *L. casei* ATCC 334, *L. reuteri* ATCC 23272, *L. paracasei* L9, *L. taiwanesis, L. plantarum* WCFS1, *L. salivarius* UCC118, *L. gasseri* SBT2055, *L. paracasei* LPC4, *L. plantarum* CLP-0611, *L. brevis* G-101, *L. plantarum* OLL2712, *L. rhamnosus* ATCC 7469, *L. rhamnosus* GG MTCC 1408, *L. helveticus* NS8, *L. rhamnosus* NutRes1, *L. plantarum* mixture (CECT 7527, CECT 7528 and CECT 7529), *L. delbrueckii* bulgaricus 2038, Intestinal *L. sp., L. rhamnosus* BFE5264 and *L. plantarum* NR74, *L. rhamnosus* OLL2838, *L. reuteri* 5289, *L. paracasei* CBA L74, *L. reuteri* DSM12246
Pro-atherogenesis	*L. rhamnosus* LA68, *L. plantarum* CJLP133, *L. brevis* HY7401, *L. rhamnosus GG ATCC 53103* & *Bifidobacterium longum* BB536, *L. fermentum* CJL-112, *L. salivarius* HMI001 & *L. casei* Shirota, *L. plantarum* nF1, *L. rhamnosus* MTCC 5897, *L. plantarum* K8, *L. acidophilus* JTB05, *L. salivarius* JTB07, *L. reuteri* JTB07, *L. acidophilus* NCFM, *L. murinus*,
Not Sure	*L. paracasei, L. rhamnosus* CRL1505, *L. casei* CHCC3137, *L. plantarum* Lb1, *L. fermentum* Lb20, *L. plantarum* 299v, *L. johnsonii* La1, *L. jensenii* TL2937, *L. rhamnosus* CNCM I-4036

Macrophages can differentiate into two subsets, pro-inflammatory subset M1 and anti-inflammatory subset M2. Some *Lactobacillus* strains can promote M1 differentiation or macrophage polarization to alter the M1/M2 ratio. The influence on pro-inflammatory production levels of macrophages is also strain-specific. Some strains like *Lactobacillus plantarum* nF1 promote pro-inflammatory cytokines secretion and other strains inhibit them. Furthermore, some strains was found to reduce ox-LDL level in human or rats.

DCs, a powerful T-lymphocyte activating factor, play a critical role in atherogenesis and can cause AS-related adverse effects. *Lactobacilli* were revealed to inhibit DC-induced inflammation and stimulate DCs to secrete anti-inflammatory cytokines like IL-10. Several studies also revealed different inflammatory responses to various *Lactobacillus* strains and experimental models, including pro-inflammatory effects.

*Lactobacilli* strains can augment NK cell activity. TLR2/NF-κB signaling pathway is involved in NK cell activity. But cytokines induced by *Lactobacilli* are strain-specific. The role of *Lactobacilli* in the atherogenesis is still unclear unless there is some direct evidence.

Similarly, the effects of *Lactobacilli* in the progression of atherogenesis via B-lymphocytes are in dispute, not only because of strain-specific immune response, but also the evidence about B-lymphocyte polarization induced by *Lactobacilli* or other direct evidence is absent.

Taken together, immune cells are very important pathways in atherogenesis, while *Lactobacilli* play their immunomodulatory effects to influence the progress of atherogenesis. But the direct study evidence, *Lactobacilli* promoting or inhibiting atherogenesis via immune cell pathways, is absent. Thus future studies are needed to explore the roles of immune cells in the atherogenesis, especially anti-atherogenetic effect, by *Lactobacillus* treatment subjects which may help to identify and properly utilize the appropriate strains.
